# Studying the pathogenicity of 26 variants characterized in the first molecular analyses of Egyptian aplastic anemia patients

**DOI:** 10.1186/s43141-023-00585-8

**Published:** 2023-11-29

**Authors:** Mona F. Sokkar, Mona Hamdy, Peter SF Erian, Rehab M. Mosaad, Nesma M. Elaraby, Mohamed B. Taher, Heba El-Sayed, Mohammed Al Komy, Maha M. Eid, Amal M. Mohamed, Khalda S. Amr, Ghada Y. El-Kamah

**Affiliations:** 1 Molecular Genetics and Enzymology Department, Human Genetics and Genome Research Institute, National Research Centre (NRC), Cairo, Egypt; 2 Department of Pediatrics, Faculty of Medicine, Cairo University, Cairo, Egypt; 3 Human Cytogenetics Department, Human Genetics and Genome Research Institute, National Research Centre (NRC), Cairo, Egypt; 4 Medical Molecular Genetics Department, Human Genetics and Genome Research Institute, National Research Centre (NRC), Cairo, Egypt; 5 Clinical Genetics Department, Human Genetics and Genome Research Institute, National Research Centre (NRC), Cairo, Egypt

**Keywords:** Aplastic anemia, Telomere, *TERT* gene, *TERC* gene, *MPL* gene, Variants

## Abstract

**Background:**

Aplastic anemia (AA) is a bone marrow disorder characterized by peripheral pancytopenia and marrow hypoplasia which can lead to life-threatening complications. Our objective was to study the telomerase genes (*TERT* and *TERC*) variants, explore their relationship to telomere shortening and *TERT* gene expression, and to identify variants in the *MPL* gene within Egyptian AA patients.

**Methods:**

Forty AA patients and 40 sex- and age-matched healthy individuals as the control group were studied through sequencing of *TERT*, *TERC*, and *MPL* genes. Quantitative real-time PCR (qRT-PCR) was used for measuring *TERT* gene expression. Telomere length (TL) was measured using the Quantitative Fluorescence In Situ Hybridization (Q-FISH) technique. In silico analysis was performed for the prediction of the pathogenicity of resultant variants.

**Results:**

Sequencing of *MPL*, *TERT*, and *TERC* genes identified 26 variants. Eleven variants were identified in the *MPL* gene. Three of them are pathogenic: two missense [c.305 G>A, c.1589 C>T] and one splice site [g.9130T>G]. *TERT* gene sequencing showed thirteen variants, among them, four novel [c.484G>A, c.499G>A, c.512G>A, c.3164C>G] and two previously reported [c.835G>A, c.2031C>T] were predicted to be pathogenic. Two variants were characterized within the *TERC* gene; n.514A>G and n.463 C>T. *TERT* gene expression was downregulated in 70% of studied patients and the Q-FISH technique detected telomere shortening in 82.5% of patients.

**Conclusions:**

Twenty-six pathogenic and benign variants within the *TERC*, *TERT*, and *MPL* genes were identified among the studied AA patients that were in several cases associated with shortened telomeres and/or lower *TERT* gene expression. Genotype/phenotype correlation in AA patients is of great importance in explaining the disease severity and guiding therapeutic decisions.

## Background

Aplastic anemia (AA) is a potentially life-threatening failure of hemopoiesis characterized by pancytopenia with hypocellular bone marrow in the absence of an abnormal infiltrate and no increase in reticulin [1]. The incidence of AA shows geographical variability varying from 10 to 52.7% among patients with pancytopenia [2] that is particularly higher in Asia and Africa. At present, the overall incidence of the disease in the general population is 3 to 6 per million, considering both inherited and acquired AA [3]. Untreated patients with AA die from cytopenia-related complications [4]. Hematopoietic stem cell transplantation (HSCT) has been developed as a therapeutic option; however, insufficient HLA-matched siblings, graft rejection, and graft versus host disease are still great challenges [5, 6]. Immunosuppressive therapy (IST) provides a better overall survival rate, but about one third of patients fail to respond [7, 8]. Relapse and clonal evolution are also obstacles in IST [9, 10]. Therefore, AA patients still have unfavorable prognoses [11].

Telomeres are heterochromatic structures with tandem DNA repeats of 5′-TTAGGG-3′ at the chromosomal ends. Telomere shortening in patients with AA was found to be significantly correlated with the degree of pancytopenia, risk of relapse after IST, clonal evolution, and overall survival [12, 13]. Telomerase is composed of two essential subunits, the human telomerase RNA component (*hTERC*), and the human telomerase reverse transcriptase *(hTERT)* catalytic component [14]. The telomerase complex adds short nucleotide repeats to the ends of chromosomes slowing the attrition rate [6].

*TERC* gene is located on chromosome 3q26.2 and comprises one non-coding exon that contains several conserved regions essential for its stability [15]. *TERT* is located on chromosome 5, and it consists of 16 exons and 15 introns spanning 35 kb, which uses the RNA component, *TERC*, as a template to catalyze telomere elongation [16].

Loss-of-function mutations in telomerase complex genes predispose to marrow failure through accelerated telomere erosion that limits the proliferative capacity of hematopoietic cells [4].

Some bone marrow failure (BMF) syndromes as congenital amegakaryocytic thrombocytopenia (CAMT) are linked to loss-of-function mutations in the *MPL* gene which encodes the thrombopoietin (TPO) receptor. CAMT is a rare inherited syndrome characterized by thrombocytopenia at birth that rapidly progresses to AA and pancytopenia [17]. The prognosis of CAMT patients is poor because all cases develop childhood trilineage marrow aplasia that is always fatal when untreated. Mutations in the *MPL* gene have been also reported in association with familial AA [18, 19].

Therefore, the current study aimed at studying the molecular pathology of different genes (*TERT* and *TERC*) involved in AA and to correlate with telomere shortening and telomerase gene (*TERT*) expression in addition to identifying variants in the *MPL* gene among Egyptian AA patients.

## Materials and methods

### Ethical statement

The study was performed following approval by the Medical Research Ethics Committee (MREC) of the National Research Center (NRC) according to the Helsinki Declaration 1975, and written informed consent was obtained from all patients or their parents

### Clinical examination of AA patients

Forty AA patients, negative for chromosomal breakage by diepoxybutane (DEB) and normal for CD55 and CD59 by flowcytometry, were recruited from the Hereditary Blood Disorders Clinic, NRC, and from Hematology Clinic, Abo El-Rish Pediatric Hospital, Cairo University. All patients were subjected to complete history taking, pedigree analyses, and thorough anthropometric evaluation, clinical examination with emphasis on hematological complaints. Five milliliters of venous blood samples were collected from patients. Blood samples were similarly collected from 40 age- and sex-matching healthy individuals as the control group. Patients were followed up after treatment with TPO agonist (Eltrombopag) and IST (cyclosporine), and responses to the treatment were assessed according to the National Institutes of Health (NIH) criteria [20].

### Molecular studies

#### Mutation screening of *TERT*, *TERC*, and *MPL* genes

DNA was extracted from the collected blood samples on EDTA using a ZYMO mini prep kit (Zymo, USA) according to the manufacturer’s instructions. PCR amplification was done using designed primers. It was performed using 10 pmol of each primer, 2mM dNTPs, 5U Taq DNA polymerase, and 100 ng DNA. PCR program was as follows: denaturation at 94ºC for 5 min, samples were then subjected to 35 cycles including (denaturation at 94ºC for 30 s, primer annealing (according to TM of each primer pair) for 30 s, and extension at 72 ºC for 30 s) followed by the final extension for 5 min at 72ºC. Purification of amplified products was done using Exonuclease I-Shrimp alkaline phosphatase (EXO-SAP) purification kit (NEB, USA). Sequencing of purified PCR products was then carried out to discover all possible genetic variations in the exonic regions and intron–exon boundaries of the telomerase genes (*hTERT* and *hTERC)* and *MPL* gene. Direct sequencing was performed in both directions via Big Dye Terminator v3.1 Cycle Sequencing Kit using an ABI Prism 3500 Genetic Analyzer (Applied BioSystems, Foster City, CA, USA). The sequences were compared to the wild-type of gene sequence retrieved from the National Center for Biotechnology Information Gene Bank (NCBI). Mutation nomenclature was given according to genetic variations approved by the Human Genome Variation Society (HGVS) (http://www.hgvs.org). Control subjects were screened for the resultant variants by Sanger sequencing.

#### RNA extraction and quantitative real-time PCR (qRT-PCR)

RNA was extracted from AA patients and controls using DirectZol RNA extraction miniprep (Zymo, USA) according to the manufacturer’s instructions. RNA was reverse transcribed to cDNA using a Cosmo cDNA synthesis kit (Cosmo, USA) and random primer according to the manufacturer’s instructions. Reverse transcription was performed under the following conditions: 2 h at 37 °C, 20 min at 80 °C. qRT-PCR was carried out to quantify the expression levels of RNA using Hera Syber green Master Mix (Cosmo, USA). Expression of the target gene *TERT* and housekeeping gene (*β actin*) have been calculated in AA cases and standardized with controls. Ct was determined for patients and controls, and then, ΔCT, ΔΔCT, and fold change expressed as relative quantification (RQ) was calculated using the following equation: ${\mathbf{R}\mathbf{Q}=2}^{-{\varvec{\Delta}}{\varvec{\Delta}}\mathbf{C}\mathbf{T}}$

### Cytogenetic studies

#### Telomere length (TL) measurement

**Fig. 1:**
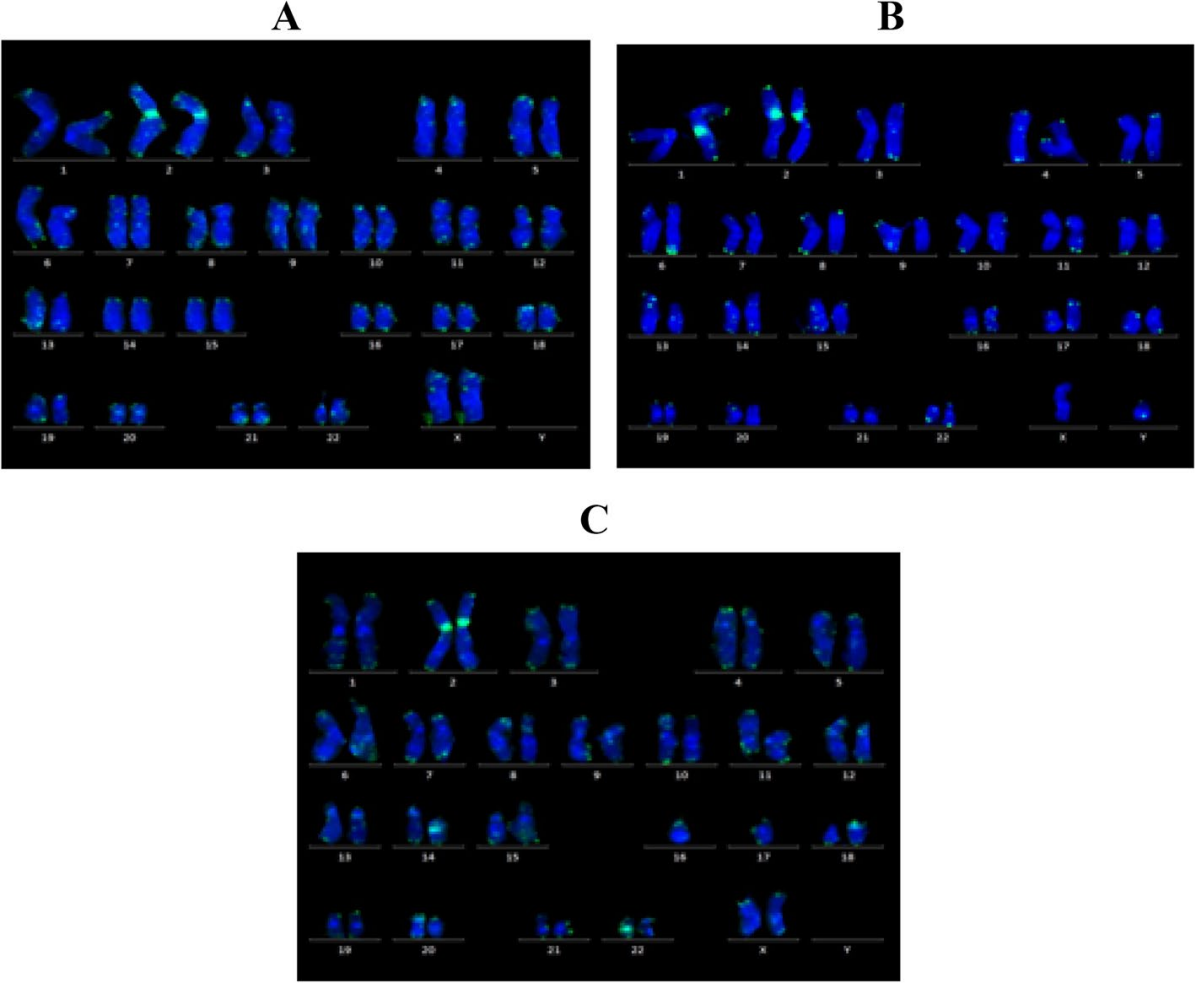
Karyotype by Q-FISH technique showing the green fluorescent signals of chromosome 2 centromere in comparison with all telomeres showing eroded signal with less fluorescence in aplastic anemia patients indicating shortened telomeres. A Aplastic anemia patient no 14. B Aplastic anemia patient no 33. C Control subject

Samples were collected on heparin. TL measurement was done for patients and controls using Q-FISH (Fig. 1). Pan Telomeres FITC-labeled and the centromeric chromosome 2 FITC-labeled probes were used to assess the TL in comparison to the reference signal of centromere 2. For each case, metaphases were examined by the analysis of TL signals for each chromosome in comparison with the reference signal (chromosome 2 centromere) resulting in a Telomere/Centromere (T/C) value. Then, the T/C values were expressed in terms of base pairs and calculated by the following equation: *y* = 2507 + 204.*x*, where *y* is the telomere length value in Kb and *x* is the T/C value calculated by the ISIS software. This equation allowed the conversion of T/C value (ratio) into base pairs in Kb, which would be expressing the actual telomere length that was compared to normal values for age [21].

### Statistical analysis

All statistical analysis was performed using the statistical software SPSS (version 20). Quantitative data was statistically represented in terms of mean and standard division (SD). Qualitative data was statistically represented in terms of number and percent. *T* test was used for comparing two parametric groups. Comparison between different groups in the present study was done using a chi-square test with odds ratio. A probability value (*P*) less than 0.05 was considered significant (*P* ≤ 0.05).

### In silico analysis

#### Sequence-based prediction

Variants with potential damaging effects (missense variants) were analyzed using in silico prediction tools, such as SIFT (http://sift.jcvi.org) and Polyphen http://genetics.bwh.harvard.edu/pph2) that categorized the detected variants into probably damaging, possibly damaging and benign. Furthermore, Mutation Tester software (http://MutationTaster.org.) was used in variants analyses where a score close to 1 indicates a prediction of the given variant to be disease-causing.

#### Structure-based prediction

Structure-based predictors, i.e., I.Mutant, MUPro mCSM, Missense3D, SDM, MAESTRO web, and PremPS, combine machine learning-based and biophysics-based approaches to determine and check the stability of mutants, calculating their free energy. The change in free energy during the unfolding of a kinetically stable protein is described by the ΔΔG value. Sometimes a single amino acid substitution in proteins differentiates the free energy landscape between the mutant and wild-type (WT) protein. This variance in the free energy landscape is the cause that the mutation affects the stability of a protein

#### Allele frequency of each variant

The frequency of all the previously reported variants was detected using the genome aggregation database (GenomAD).

## Results

### Clinical results

Forty aplastic anemia cases (29 males and 11 females) were included in the study based on their peripheral hemogram and Bone marrow cellularity (Bone marrow aspirate and biopsy) (BMA and BMB). According to modified Camitta criteria [22], 30/40 (75%) were categorized as severe AA. The mean age for patients was 8.01± 4.07 years. All patients were negative for chromosomal breakage by DEB and had normal CD55 and CD59 by flow cytometry.

Twenty-one patients were descending from consanguineous pedigrees. Five patients had a positive family history of AA. Two patients were clinically classified as Dyskeratosis Congenita (DC) (patients no 9 & 10). Anthropometric evaluation detected microcephaly in five patients and short stature in five patients. Several anomalies were also detected through clinical evaluation including skeletal affection in the form of absent ulna and radius in one patient, undescended testis in two patients, neurological affection in three patients, ventricular septal defect (VSD) in one patient, renal anomaly (absent Lt kidney) in one patient, and skin pigmentation including café au lait patches in four patients. Re-evaluation of patients following treatment revealed that 22/40 (55%) did not exhibit any therapeutic response, 11 cases (27.5%) showed good response, and 5 patients spontaneously recovered prior to treatment. The DC patients did not receive treatment and did not undergo HSCT. Forty healthy individuals (25 males and 15 females) were enrolled in the study as the control group with a mean age of 9.32 ± 3.21.

Phenotype–genotype correlation of AA patients with detected variants was summarized in Table 1.

**Table 1:** Genotype/phenotype correlation in AA patients

**No**	**Sex**	**Age/year**	**Consanguinity**	**Family history**	**Hb g/dl**	**WBCs Cells x 10**^**3**^	**Plts Cells/cm**^**3**^	**BM cellularity %**	**Severity**	**Clinical presentation**	**Associated abnormalities**	***MPL* gene**	***TERT* gene**	***TERC* gene**	***TERT* expression**	**Telomere length**	**Follow-up**
1	M	11	+	-	9	4	95,000	40–50	Moderate	Pallor	Short stature Microcephaly Ventricular septal defect (VSD) Skin pigmentation Neuroglial manifestation Undescended testis	-	c.835G>A , hetero/c.761G>A, hetero	n.514 A>G, hetero	Downregulated	Shortened	No response
2	M	9.5	+	-	6.4	3.6	80,000	<10	Severe	Pallor-Ecchymosis	Neurological manifestation (CP) Microcephaly short stature Undescended testis	-	c.835G>A, hetero/c.1336C>, hetero	-	Downregulated	Shortened	No response
3	M	3	+	-	5,9	4	10,000	5–10	Severe	Pallor, Petechiae	Dysmorphic facies-microcephaly	g.11634T>A, hetero	-	n.514 A>G, homo	Normal	Shortened	No response
4	M	8	+	_	8	3.5	40,000	40–50	Moderate	Petechiae-ecchymosis		-	c.512G>A, hetro	-	Downregulated	Shortened	Response
5	F	4	-	-	10.4	5.4	17,000	<10	Severe	Petechiae ecchymosis	-	g.11634T>, hetero	c.915G>A, hetero	n.514 A>G, hetero	Downregulated	Shortened	Good response
6	M	16	+	+	9.3	3.6	17,000	<10	Severe	Weight loss-fatigue-petechiae	-		Double hetero, c.835G>A /c.2031C>T	-	Downregulated	Shortened	Relapse and Develop myelodysplasia
7	M	10	+	-	6	3.2	16,000	<10	Severe	Ecchymosis–hematemesis syncopal attacks	-	g.11634T>, hetero	c.512G>A hetero/c.915G>A,hetero	-	Downregulated	Normal	Good response
8	M	9	+	-	7	3	10,000	10–20	Severe	Ecchymosis-epistaxis	-	c.543T>C hetero	c.512G>A hetero g.22755A>G, hetero,	-	Downregulated	Shortened	Good response
9	M	13	+	+	10	4.2	47,000	40–50	Moderate	Recurrent infection and fever	Abnormal skin pigmentation, nail dystrophy Microcephaly - shot stature	g.11634T>, hetero	c.915G>A, hetero	-	Downregulated	Shortened	No treatment Undergo HSCT
10	M	7	+	+	9.5	4	40,000	40–50	Moderate	Recurrent infection and fever	Abnormal skin pigmentation - nail dystrophy	g.11634T>, hetero	c.915G>A, hetero	-	Downregulated	Shortened	No treatment Undergo HSCT
11	M	7	+	-	9	3.2	37,000	<10	Severe	Pallor-petechiae -ecchymosis	Neurological manifestation (hyperreflexia of LL)	-	c.2031C>T, hetero	-	Downregulated	Shortened	No response
12	M	3	+	+	9.1	6.8	10,000	<10	Severe	Epistaxis-bleeding gums-hematemesis	-	c.1589 C>T homo	c.499G>A, hetero		Downregulated	Shortened	No response
13	M	4	-	-	10.3	4.6	21,000	<10	Severe	Pallor-Jaundice	-	-	c.499G>A, hetero	n.463 C>T hetero	Normal	Shortened	Good response
14	F	3	-	-	7.8	3	13,000	10–20	Severe	Epistaxis-subconjunctival hemorrhage	-	c.305G>A, homo	c.499G>A, hetero/c.915G>A, hetero	-	Normal	Shortened	No response
15	M	11	+	-	6.4	3.2	12,000	<10	Severe	Fever-bleeding gums	-	-	-	n.514 A>G, hetero	Normal	Shortened	No response
16	F	13	-	-	8	2.4	11,000	30–40	Moderate	Purpura-ecchymosis	-	g.11387T>C, hetero	-	n.514 A>G, hetero	Downregulated	Normal	No response
17	F	3	+	-	8	7.4	15,000	<10	Severe	Pallor	Short stature renal anomaly (absent LT kidney)	g.9130T>G, hetero	c.835G>A, hetero	-	Downregulated	Shortened	No response
18	M	6	-	-	9	1.8	2000	<10	Severe	Bleeding gums-bruises Ecchymosis	-	g.11387T>C, hetero	c.484 G>A, hetero c.915G>A, hetero g.40734A>G, hetero	-	Downregulated	Shortened	No response
19	M	11	+	-	8.9	1.8	10,000	<10	Severe	Bleeding per gums-ecchymosis	Intracranial hemorrhage-delayed mental development	-	-	n.514, A>G, hetero	Downregulated	Marked shortening	No response
20	M	3	-	+	5	3.9	90,000	40–45	Moderate	Severe pallor	-	-	-	n.514 A>G, hetero	Downregulated	Shortened	No ttt
21	M	3	-	-	8	3.4	21,000	<10	Severe	Petechiae -ecchymosis	-	c.340G>A, hetero	c.3164C>G, hetero/c.915G>A, hetero	-	Normal	Shortened	No ttt
22	F	12	+	-	8	2.9	40,000	<10	Severe	Epistaxis, bruises	Microcephaly	-	c.499G>A hetero	-	Downregulated	Shortened	Response
23	M	5	-	-	10.5	4.7	108,000	30–40	Moderate	Spontaneous ecchymosis	-	-	c.499G>A hetero	-	Normal	Shortened	Response
24	M	10.5	-	-	9.4	1.8	25,000	<10	Severe	Purpuric eruption	-	c.401C>G, hetero\g.8598G>A hetero, g.11387T/C, hetero	-	-	Downregulated	Shortened	No ttt
25	M	10	-	-	6	3	5000	<10	Severe	Pallor petechiae	-	-	c.499G>A, hetero c.915G>A, hetero	-	Downregulated	Marked shortening	No response
26	M	15	-	-	8	3.5	13,000	<10	Severe	Petechiae	café au lait patches	g.11634T>, hetero		-	Normal	Shortened	Good response
27	F	7	-	-	10.5	3.2	70,000	<10	Severe	Petechiae-recurrent infection	-	-	-	-	Downregulated	Shortened	Good response
28	M	8	-	-	9.4	4.6	34,000	<10	Severe	Petechiae ecchymosis	-	g.11634T>A, hetero	-	-	Downregulated	Shortened	Good response
29	M	14	+	-	7	3	12,000	10–20	Severe	Pallor-Easy fatigability	-	c.1631G>A, hetero g.11634T>A, hetero	-	-	Normal	Shortened	No response
30	M	3	-	-	6.1	3.2	92,000	<10	Severe	Fever - sepsis	-	g.11634T>A, hetero	-	-	Downregulated	Shortened	No response
31	M	6	-	-	7	1.7	5000	<10	Severe	Purpura-ecchymosis	-		c.690A>G hetero	-	Normal	Shortened	No response
32	M	12	+	-	8.7	4.9	37,000	<10	Severe	Pallor-petechiae	-		g.40734A>G	-	Normal	Normal	No response
33	M	9	+	-	7.5	4.4	50,000	<10	Severe	Pallor-epistaxis	-		c.915G>A, hetero	-	Normal	Shortened	No response
34	F	10	+	-	7.8	9	68,000	<10	Severe	Petechiae	-	-c.340G>A, hetero	-	-	Downregulated	Shortened	No response
35	F	5	-	-	7.2	1.4	50,000	<10	Severe	Ecchymosis-petechiae-epistaxis-bleeding from other orifices	-	g.11387T>C, hetero	915G>A, hetero -	-	Downregulated	Shortened	No response and died from sepsis
36	F	12	+	-	8.2	2.2	28,000	<10	Severe	Menorrhagia-vaginal bleeding-epistaxis	-	g.11387T>C, hetero	-	-	Downregulated	Normal	No response
37	M	1.5	-	-	11.2	6	25,000	40	Moderate	Petechiae	Absent ulna and radius	-	c.915G>A, hetero	-	Downregulated	Normal	No ttt
38	F	6.5	+	-	9.5	4.9	39,000	30–40	Moderate	Pallor-Anemia	-	-	-	-	Downregulated	Normal	No ttt
39	M	12.5	-	-	7.4	3.5	6000	<10	Severe	Pallor-dizziness-fatigue	-	c.340G>A, hetero	g.22912G>T, hetero	-	Downregulated	Shortened	Good response
40	F	13	-	-	9	3.8	90,000	40–50	Moderate	Pallor	Short stature-underweight Hypoplastic nose	g.11634T>A, hetero	-	-	Normal	Normal	No response

### Molecular results

All variants detected in the present study and their frequency in (GenomAD) were summarized in Table 2.

**Table 2:** List of variants detected in *MPL*, *TERC*, and *TERT* genes

**Affected gene**	**Exon/Intron**	**Nucleotide change**	**Amino acid change**	**GenomAD frequency**	**Variation type**	**Status of variant/no of pts**	**Reference dbSNP ID**	**Reference**
*C-MPL *NM_005373.3	**Exon 3**	g.828G>A (c.305G>A)	p.Arg102His	0.00000795	Pathogenic	Homozygous/1	rs28928907	[23]
		g.863G>A(c.340G>A )	p.Val 114Met	0.0246	Benign	Heterozygous/3	rs12731981	[24]
	**Exon 4**	g.1474C>G (c.401C>G)	P.Ala143Gly	Not detected	Benign	Heterozygous/1	Novel	**--------**
		g.1616T>C (c.543T>C)	P.Gly181=	0.00202	Benign	heterozygous/1	rs17572791	[25]
		g.1763A>G (c.690A>G)	p.Glu 230=	0.0344	Benign	heterozygous/1	rs16830693	**Reported in Clinvar**
	**Intron 6**	g.8598G>A (c.981–41G>A)	Intronic	0.312	Benign	Heterozygous/1	rs1760670	**Reported in Clinvar**
	**Intron 8**	g.9130T>G(+81T>G)	Splice site	Not detected	Benign	Heterozygous/1	Novel	**------**
	**Intron 9**	g.11387T>C(c.1469–70 T>C)	Intronic	0.301	Benign	Heterozygous/4, homozygous/1	rs839995	**Reported in dbSNP **
	**Intron 10**	g.11634T>A	-------	Not detected	Benign	Heterozygous/10	Novel	**------**
	**Exon 11**	g.14433C>T (c.1589 C>T)	P.Pro530 leu	0.00000797	Pathogenic	Homozygous/1	Novel	**----**
		g.14475G>A(c.1631G>A)	p. Arg544Lys	Not detected	Benign	Heterozygous/1	Novel	**-----**
*TERC* NR_001566.1	**Down-stream**	n. 514A>G	-----	0.00000675	Benign	Heterozygous/6, Homozygous/1	rs2293607	[26]
	**3 prime UTR**	n. 463C>T	------	0.000119	Benign	Heterozygous/1	rs150338214	**Reported in Clinvar**
*TERT* NM_198253.3	**Exon 2**	g.668 G>A ( c.484 G>A)	p.Val162Met	Not detected	Pathogenic	Heterozygous/1	Novel	-----
		g.683G>A (C.499G>A)	p.Ala167Thr	Not detected	Conflicting	Heterozygous/6	Novel	-----
		g.696 G>A (c.512G>A)	p.Cys171Tyr	Not detected	Pathogenic	Heterozygous/3	Novel	-----
		g.945 G>A ( C.761G>A)	P.Gly254Glu	Not detected	benign	Heterozygous/1	Novel	-----
		g.1019 >/A (c.835 G>A)	p.Ala279Thr	0.0225	conflicting	Heterozygous/4	rs61748181	[26]
		g.1099>/A (c.915G>A)	p.Ala305=	0.287	benign	Heterozygous/9,Homozygous/2	rs2736098	[27]
		g..1520C>G(c.1336C>G)	P.Arg446Gly	0.000841	Benign	Heterozygous/1	rs567650961	**Reported in Clinvar**
	**Exon 5**	g.15680C>T (c.2031C>T)	p.Gly677=	0.0101	Conflicting	Heterozygous/2	rs33956095	[27]
	**Intron 4**	g.15579C>G(+21C>G)	Intronic	Not detected	Benign	Heterozygous/1	Novel	**-------**
	**Intron6**	g.22755A>G(+35A>G)	Intronic	Not detected	Benign	Heterozygous/1	Novel	**-------**
	**Intron 7**	g.22912G>T(c.2382+27G>T	Intronic	0.000192	Benign	Heterozygous/1	rs200578375	**Reported in dbSNP**
	**Exon 15**	g.40571C>G(c.3164C>G)	P.Ser1055Trp	Not detected	Pathogenic	Heterozygous/1	Novel	**------**
	**Intron 15**	g.40734A>G(c.3295+32A>G	Intronic	0.0151	Benign	Heterozygous/2	rs34742644	[28]

*GenomAD* genome aggregation database, *dbSNP* single-nucleotide polymorphism database

#### Sequencing of *MPL* gene

**Fig. 2:**
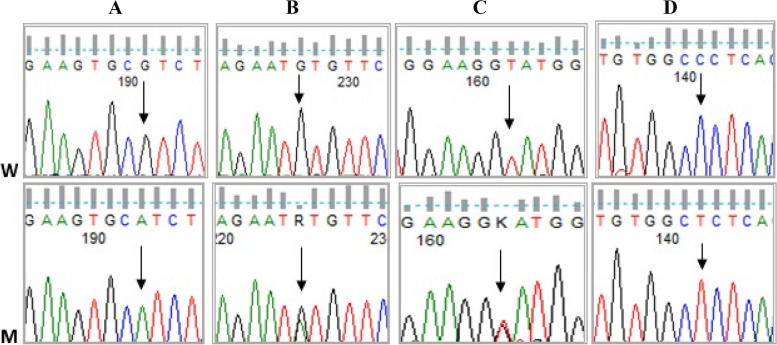
Partial nucleotide sequence of [NM_005373.3, *MPL* gene]. **A** Variant c.305G>A in exon 3. **B** Variant c.340G>A in exon 3. **C** Variant g.9130T>G in intron 8. **D** Variant c.1589C>T in exon 11 (W for wild and M for mutant)

Eleven variants were identified in the *MPL* gene. Exon 3 harbored two previously reported variants. [c.305G>A] was found in a homozygous state in one patient (2.5%), and in silico analysis indicated that it is likely pathogenic. The second variant [c340G>A] was detected in a heterozygous state in three cases (7.5%) (Fig. 2).

Additionally, three benign variants c.401C>G, c.543T>C, and c.690A>G were identified in exon 4 of the *MPL gene*, among which c.401C>G was a novel unique variant that was not reported in various SNP databases. The variants [c.543T>C and c.690A>G] were previously reported and had low frequencies of 0.00202 and 0.0344 on GenomAD.

Another two novel exonic variants [c.1589 C>T and c.1631G>A] were detected in exon 11. (C.1589 C>T) was detected in a homozygous state in one patient. In silico analysis indicated that it is likely to be pathogenic; however, the other variant [c.1631G>A] was predicted to be likely benign. Also, a novel splice site variant [g.9130T>G] was detected in one patient and predicted to be pathogenic by in silico analysis (Fig. 2).

Three intronic variants were detected: g.8598G/A, g.11387T>C, and g.11634T>A. Interestingly, g.11634T>A was a novel benign associated variant that was found in ten patients (25%) in a heterozygous state and was not detected within the control group. Considering the control group, only three *MPL* variants were detected: c.690 A>G, g.8598G/A, and g.11387T>C, each in one control subject.

#### Sequencing of *TERT* gene

Thirteen variants were detected in the *TERT* gene. Seven missense variants were detected in exon 2: four novel variants [c.484 G/A, c.499G>A, c.512G>A,c.761G>A] and three previously reported [c.835G/A, c.915G>A, c.1336C/G]. In silico analysis of these variants predicted that [c.484G/A, c.512G>A] is likely pathogenic and [c.761G>A, c.1336C>G] is likely benign.

The variant [c.499G>A] had conflicting pathogenicity. It was identified in six patients (15%) in heterozygous state. Also, the previously reported variant [c.835G>A] showed conflicting pathogenicity. It was characterized by a heterozygous state in 4 cases (10%) and one control (2.5%). The other previously reported benign variant [c.915G>A] was identified in eleven patients (27.5%), 9 patients in a heterozygous state, and 2 patients in a homozygous state.

**Fig. 3:**
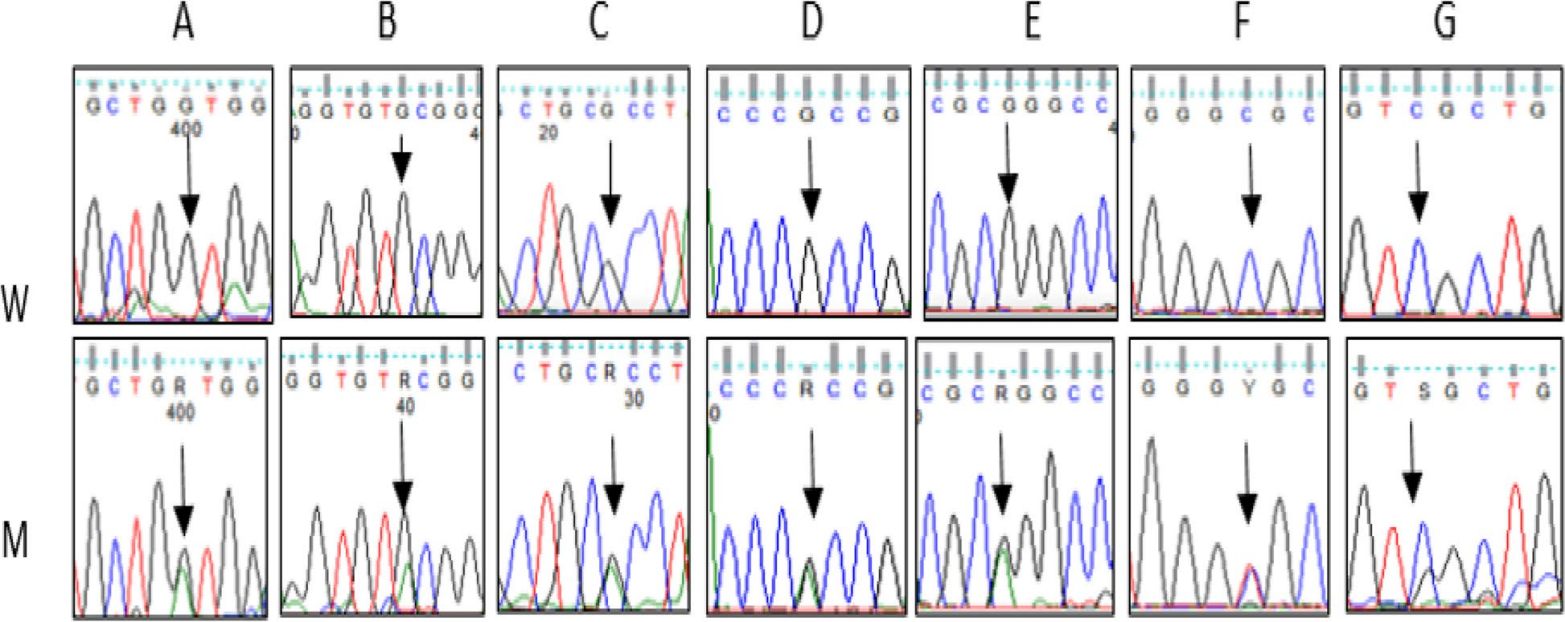
Partial nucleotide sequence of [NM_198253.3, *TERT* gene]. **A** Variant c.484G>A in exon 2. **B** Variant c.512G>A in exon 2. **C** Variant c.499G>A in exon2. **D** Variant c.835G>A in exon2. **E** Variant c.915G>A in exon 2**. F** Variant c.2031C>T in exon 5. **G** Variant c.3164C>G in exon 15 (W for wild and M for mutant)

A previously reported synonymous variant [c.2031 C>T] was identified in exon five of the *TERT* gene. It was found in a heterozygous state in two patients (5%). It had conflicting pathogenicity and had low frequency (0.0101) on GenomAD (Fig. 3).

Furthermore, we found a novel missense variant [c.3164 C>G] in exon 15 that was not previously reported in mutation databases. It was identified in a heterozygous state in one patient and predicted to be likely pathogenic (Fig. 3).

Four associated intronic variants were also detected: two novel variants [g.15579C>G and g.22755A>G] that were not previously reported in SNP databases and two previously reported variants [g.22912G>T and g.40734A>G] whose frequencies on GenomAD were 0.000192 and 0.0151, respectively. Investigating the control group for *TERT* variants revealed that only one control carried the variant [c.835G>A].

#### Sequencing of *TERC* gene

**Fig. 4:**
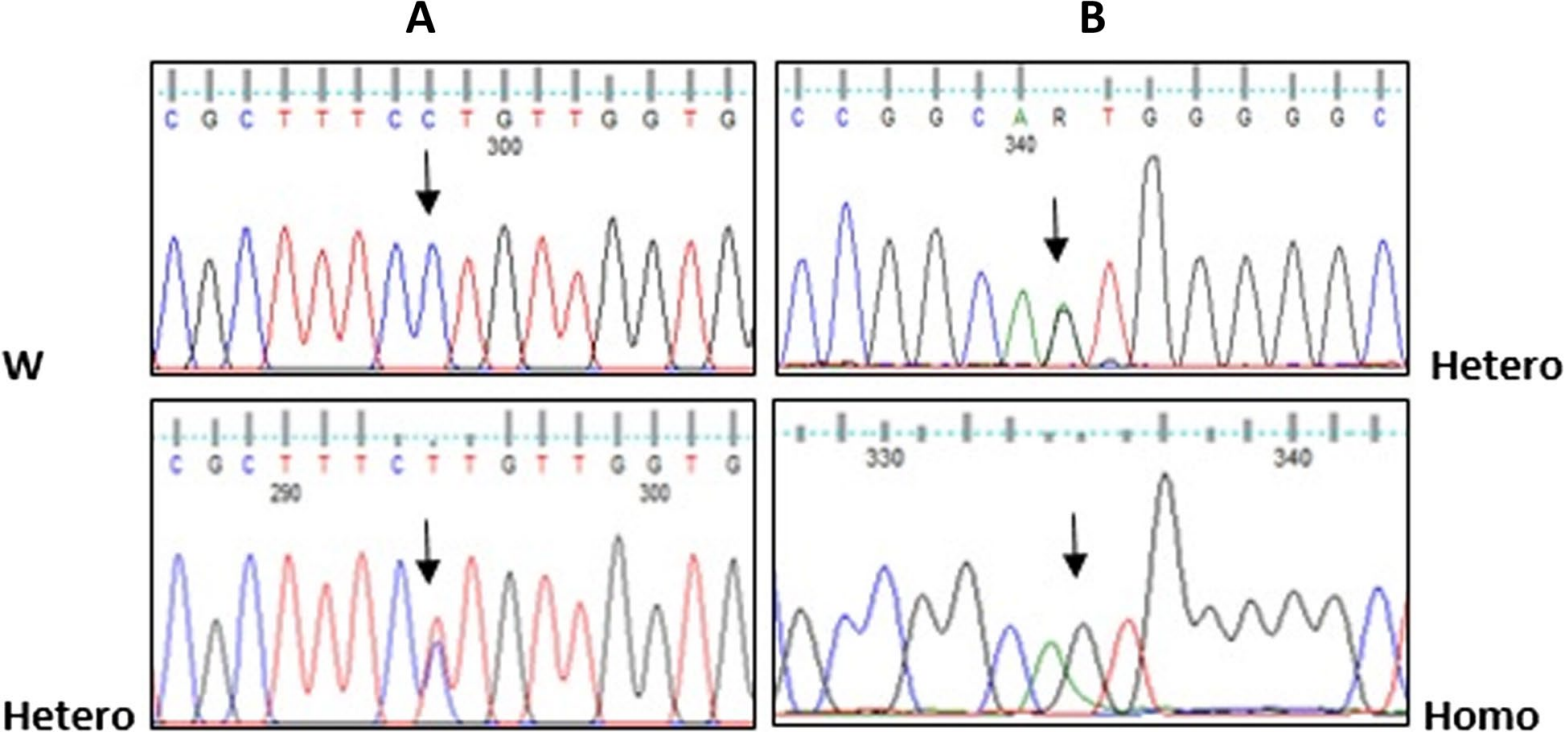
Partial nucleotide sequence of [NR_001566.1, *TERC* gene]. **A** Variant n.463 C>T. **B** Variant n.514 A > G (W for wild, Hetero for heterozygous and Homo for homozygous)

Molecular characterization of the *TERC* gene identified two variants: (n.514 A>G) variant rs2293607 was identified in 7 patients (17.5%) (one patient had the variant in homozygous state and 6 patients in heterozygous state). The other variant (n.463 C>T) rs150338214 was detected in one patient in a heterozygous state (Fig. 4). The two variants have low frequencies on GenomAD which were 0.00000675 and 0.000119, respectively, and did not detect in the healthy participants.

### Results of *TERT* gene expression analysis

**Fig. 5:**
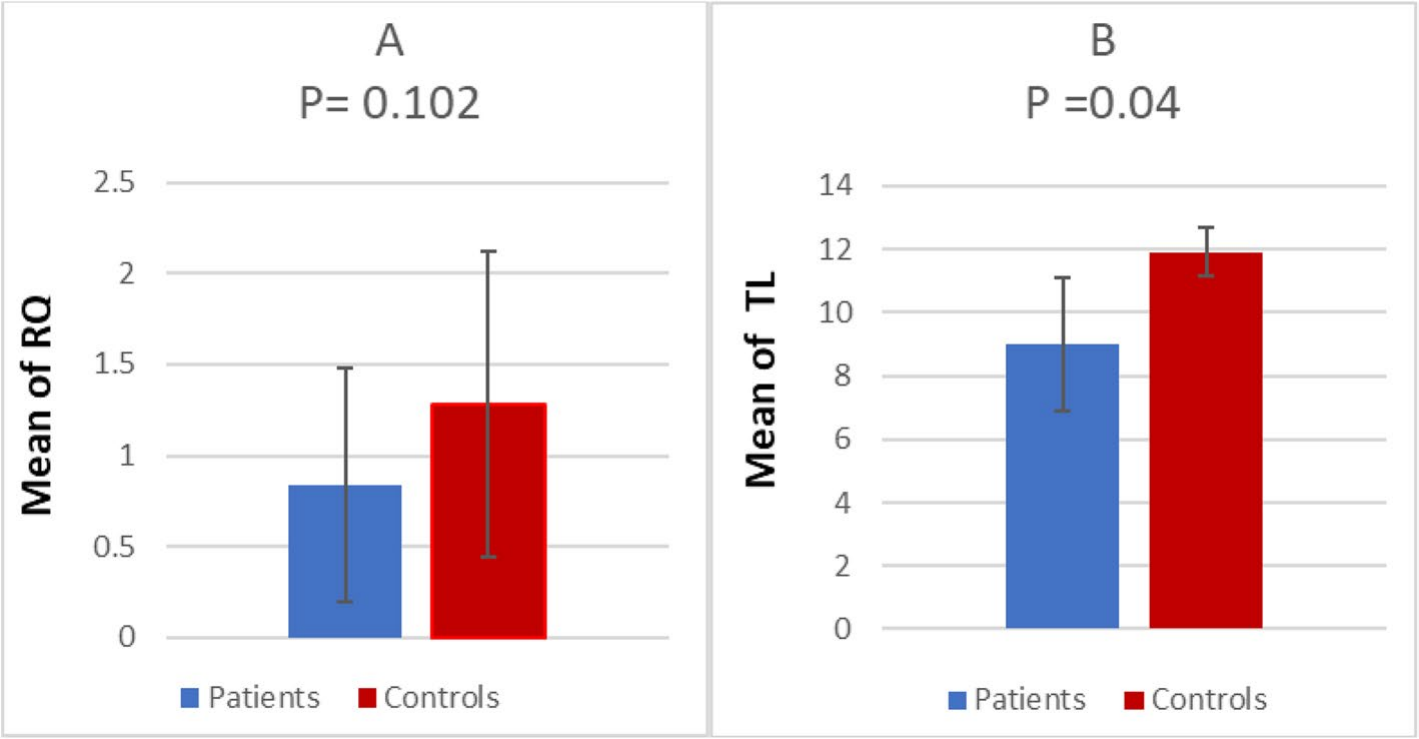
Comparison between patients and controls. **A** Comparison of RQ of *TERT* gene expression. **B** Comparison of TL. *P*≤ 0.5 is considered significant

Calculated “RQ” of *TERT* gene expression in the studied groups showed that 70% of AA cases had lower expression of the *TERT* gene. Although the mean for RQ of *TERT* gene expression was lower in AA patients than in controls, it did not reach a statistical significance (*P*=0.102) (Fig. 5).

No significant variance was identified between *TERT* gene expression and therapeutic response (*P*=0.25) or TL (*P*=0.093).

### Cytogenetic results

Measuring the TL in all patients revealed that 82.5% of cases had short telomeric length according to the reference normal value for age. In comparison to control subjects, TL was statistically shorter in AA cases (8.979 ±2.088) than in the studied controls (11.916 ± 0.76459) (*P*=0.04) (Fig. 5).

Among our investigated patients, two patients with severe AA (pt .no 19 & 25) had marked telomere shortening with telomere length (2.9 and 3.2 kb) in relation to normal values for age (10.1–11.1 kb). These two patients were noticed to be irresponsive to therapy.

There was not a statistically significant difference between the TL and the therapeutic response (*p*>0.05).

### In silico analysis

The in silico analysis tools used for the prediction of the pathogenicity of variants are illustrated in (Table 3). Regarding protein stability, in silco analysis was also carried out to indicate the protein stability changes based on reliability index (RI) scores. All analyzed missense variants except [c.401>/G] of the *MPL* gene and [c.761G>A] of the *TERT* gene had ΔΔG <0 indicating that these variants are destabilizing and predicted to result in a decrease in protein stability, which is suggestive to cause alteration in proteins’ structure (Table 4).

**Table 3:** In silico analysis of missense variants in target genes

Gene	**Missense variants**	**GenomAD frequency**	**dbSNP ID**	**SIFT**	**Polyphen-2** **HumDiv**	**Mutation taster**
***C-MPL *NM_005373.3**	c.305G>A	0.00000795	rs28928907	Tolerated	Probably damaging with a score of 0.97 (sensitivity: 0.76; specificity: 0.96)	Disease-causing
	c.340G/A	0.0246	rs12731981	Tolerated	Probably damaging with a score of 0.996 (sensitivity: 0.55; specificity: 0.98)	Polymorphism
	c.401C>G	Not detected	Novel	Tolerated	Probably damaging with a score of 0.008 (sensitivity: 0.96; specificity: 0.48)	Polymorphism
	c.1589C>T	0.000797	Novel	Damaging	Probably damaging with a score of 1.000 (sensitivity: 0.00; specificity: 1.00)	Disease-causing
	c.1631G>A	Not detected	Novel	Tolerated	Benign score 0.015 (sensitivity: 0.69; specificity: 0.79)	Polymorphism
***TERT* NM_198253.3**	c.484G>A	Not detected	Novel	Damaging	Probably damaging with a score of 0.99 (sensitivity: 0.14, specificity: 0.99)	Disease-causing
	C.499G>A	Not detected	Novel	Tolerated	Probably damaging with a score of 0.91 (sensitivity: 0.81; specificity: 0.94)	Polymorphism
	c.512G>A	Not detected	Novel	Damaging	Possibly damaging score 0.61 (sensitivity: 0.87; specificity: 0.91)	Disease-causing
	C.761G>A	Not detected	Novel	Tolerated	Benign score 0.118 (sensitivity: 0.93; specificity: 0.86)	Polymorphism
	c.835 G>A	0.0225	rs61748181	Tolerated	Probably damaging with a score of 0.98 (sensitivity: 0.75; specificity: 0.96)	Disease-causing
	c.1336C>G	0.000841	rs567650961	Tolerated	Benign score 0.021 (sensitivity: 0.95; specificity: 0.80)	Polymorphism
	c.3164C>G	Not detected	Novel	Damaging	Probably damaging with a score of 0.99 (sensitivity: 0.14; specificity: 0.99)	Polymorphism

*GenomAD* genome aggregation database, *dbSNP* single-nucleotide polymorphism database

**Table 4:** The protein stability in missense variants

**Gene**	**Nucleotide change**	**AA** **Wt/new**	**Stability**	**RI**	ΔΔG **kcal/mol**
***MPL gene***	c.305G>A	Arg/His	Decrease	9	−1.442
	c.340G>A	Val/Met	Decrease	7	−2.37
	c.401C>G	Ala/Gly	Increase	2	0.17
	c.1589 C>T	Pro/leu	Decrease	3	−0.209
	c.1631G>A	Arg/Lys	Decrease	8	−0.02
***TERT gene***	c.484G>A	Val/Met	Decrease	7	−0.8
	c.499G>A	Ala/Thr	Decrease	9	−2.12
	c.512G>A	Cys/Tyr	Decrease	7	−0.78
	C.761G>A	Gly/Glu	Increase	9	0.21
	c.835 G>A	Ala/Thr	Decrease	8	−0.682
	c.1336C>G	Arg/Gly	Decrease	8	−0.60
	c.3164C>G	Ser1/Trp	Decrease	8	−0.31

*AA* Amino acid, *wt* Wild type, *New* amino acid after mutation, *RI* Reliability index, *ΔΔG* predicted stability change

## Discussion

Aplastic anemia is a life-threatening hematopoietic disease characterized by peripheral pancytopenia accompanied by BM aplasia. AA may develop at any age of life [29]. In the current research, we investigated telomerase (*TERT*, *TERC*) and *MPL* genes for variants.

The *TERC* gene is located on chromosome 3q26.2. It codes for RNA components of telomerase and maintains telomerase catalytic activity [30, 31].

In the current study, we found two previously reported variants in the *TERC* gene. (n.514A>G) were detected in 7 cases (17.5%) and were not detected in any of the examined controls. This variant is caused by A→G substitution at the 3′ downstream region. Polat et al. [32] suggested that it might change the secondary mRNA structure of the transcript by folding the RNA. Different results were found by [26] who examined 96 AA patients and 76 healthy subjects in Japan and detected this variant in 59% of patients and 53% of controls. This higher prevalence might be attributed to the ethnic variability and the larger sample size studied.

Another benign variant (n.463C>T) located at 3′ UTR was detected in one patient in a heterozygous state who had shortened TL and experienced a good response to IST. We observed that the two *TERC* variants had low frequencies on GenomAD and were not identified in the control group indicating the possible pathogenic role of these variants.

We also investigated the *TERT* gene, which encodes telomerase reverse transcriptase, and our results revealed thirteen variants.

A previously reported variant c.835G>A was identified in 4 cases (10%) and one control (2.5%) in a heterozygous state. This variant is located in exon 2 in a linker domain of the N-terminal region of the *TERT* gene and led to amino acid change p. Ala279Thr. The cases that carried this variant also exhibited low *TERT* expression and short TL and had poor response to IST which indicated that this variant may have a role in the pathogenesis of AA. In line with our results, the same findings were reported by Liang et al. [26]. In contrast, Du et al. and Yamaguchi et al. [33, 34] demonstrated that (G835A) has been reported also in control subjects with similar allelic frequency and concluded that c.835G>A was a SNP and unlikely to cause BMF. This controversy regarding the significance of this variant was also reported by Akram et al. [35].

Additionally, three novel missense variants were detected in exon 2 of the *TERT* gene. The first variant [c.484 G>A], which causes p. Val162Met, was found in one patient having shortened telomere. The other novel variant c.512G>A (p.Cys171Tyr) was characterized in three patients (7.5%). Both variants are predicted to be pathogenic and likely to cause a decrease in protein stability, which suggestively causes a greater loss of the *TERT* protein that was confirmed in the present study by decreased *TERT* expression in these patients. Although the patients carrying these two variants presented with severe phenotypes, they did not respond to the therapy in the same way. The patient carrying [c.484 G>A] did not respond to IST while a good response to therapy was demonstrated in the patient carrying [c.512G>A].

The third novel variant [c.499G>A] with conflicting pathogenicity was detected in six patients (15%) in a heterozygous state. All carriers of this variant had short telomeres.

Screening of exon 5 of the *TERT* gene identified one synonymous variant [c.2031 C>T]. It was found in two patients (5%) in heterozygous state. Similarly, Vulliamy et al. [27] studied eighty BMF patients in London and detected this variant in 3.7% of the studied patients. Despite having conflicting pathogenicity, this variant had a low frequency on GenomAD and was not identified in the studied controls.

Additionally, one patient (2.5%) with shortened telomeres exhibited a novel missense mutation [c.3164 C>G] in exon 15 which was anticipated to be pathogenic. Interestingly, our results reported that one patient, who was a compound heterozygote for two *TERT* gene variants (c.835G>A/c.2031C>T), suffered relapse and development of myelodysplasia following therapy. This patient also showed short telomeric length and decreased *TERT* gene expression confirming the potential pathogenicity of these variants.

Furthermore, seven benign variants were identified within the *TERT* gene, c.761G>A, c.915G>A, c.1336C>G, g.15579C>G, g.22912G>A, g.22755A>G, and 40734A>G.

Among them, c.915 G>A was detected in eleven (27.5%) AA patients with short telomeres but not in the healthy participants implying that this variant might be genetically linked to telomere shortening in AA patients. This could be brought about by its location within the gene regulatory elements and alteration of transcription factor binding [36]. In agreement with our results, Vulliamy et al. [27] identified 16/80 (20%) of BMF patients as carriers of the c.915G>A variant. According to previous studies, this variant showed a significant association with cancer risk [37, 38]. Thus, it could be considered as a marker for cancer development in AA cases to be examined for early diagnosis and management using targeted cancer therapy [39, 40].

*MPL* gene was also screened in the present study, due to its important role in the development of megakaryocytes and platelets as well as the self-renewal of hematopoietic stem cells [41]. Loss-of-function mutations in the *MPL* gene can be directly linked to BMF syndromes such as CAMT, a rare inherited syndrome characterized by thrombocytopenia at birth that rapidly progresses to AA and pancytopenia [17].

The current research identified eleven different variants in the *MPL* gene. A likely pathogenic variant c.305 G>A, that results in aa substitution (p. Arg102 His), was characterized in a homozygous state in one patient with short TL and presented to our clinic with marked thrombocytopenia and unresponsiveness to IST. Similar in silico analysis results were reported by Germeshausen and Ballmaier [23].

A novel missense variant [c.1589 C>T] that are predicted to be pathogenic was identified in one of our investigated patients in a homozygous state. This variant led to amino acid change Pro530leu, and the patient harboring this variant had severe AA, short telomere and was refractory to therapy.

Moreover, another novel splice site variant g.9130T>G was detected in one patient with short telomere and marked thrombocytopenia. This variant lies 2bp downstream exon 8 and alters the normal splicing. This type of mutation may cause complete loss of function in the TPO receptor as mentioned by Germeshausen et al. [42] who investigated the *MPL* gene in 23 German patients with CAMT and found two splice site variants [c.79+2T>A and c.213-1G>A].

We also identified eight associated variants within the *MPL* gene, c.340G>A, c.401C>G, c.543T>C, c.690 A>G, g.8598G>A, g.11387 T>C, c.11634T>A, and c.1631G>A.

The variant, c.340G>A, being reported as an underlying cause for CAMT, was detected in three of our AA cases who presented with marked thrombocytopenia and was not found in the control group. In contrast, Oudenrijn et al. [24] mentioned that *MPL* G340A which leads to amino acid change p. Val114Met and affects the extracellular portion of the TPO receptor has been detected in CAMT patients as well as healthy subjects.

Additionally, the abovementioned novel variant [g.11634T>A] was proposed to be AA associated variant being detected in ten patients (25%) but not in the control group.

In the current research, studying the *TERT* gene expression revealed that it was downregulated in 70% of AA patients. In accordance with our results, Gupta et al. [43] observed that 17/24 (70.8%) idiopathic AA patients exhibited 8-fold reduction in *TERT* gene expression and suggested that loss of *TERT* expression correlates with disease severity and poor prognosis.

Moreover, cytogenetic studies in the current study revealed shortened telomeres in 82.5% of the studied cases with significant variance between TL in patients and controls (*P*=0.04). Additionally, marked telomere shortening was observed in two patients with severe AA who were irresponsive to therapy.

Many previous studies also reported telomere shortening in 30–50% of studied individuals with AA [43, 44]. Accelerated telomere shortening might result from defects in telomerase that prematurely limit the proliferation potential of cells, including stem cells, leading to decreased tissue renewal capacity [45] with a possible correlation to the rapid turnover of hematopoietic progenitor cells to compensate for BMF [16].

In the current investigation, no significant variance was found between TL and the presence of telomerase gene mutations or *TERT* gene expression. This may be attributed to the phenomenon of disease anticipation and that many of the *TERC* and *TERT* mutations are hypomorphic, impairing but not abolishing telomerase activity [33].

Furthermore, TL was not statistically associated with therapeutic response (*p*>0.05). Consistent results were reported by Scheinberg et al. [12] who concluded that TL in severe AA cases receiving IST was not associated with response, but was associated with risk of relapse, clonal evolution, and overall survival. He also suggested that an insufficient number of hematopoietic stem cells, inadequate immunosuppression, and a nonimmune etiology for BMF may be implicated in that observation.

In conclusion, reviewing the literature, this is the first molecular study in Egypt to screen collectively *MPL*, *TERC*, and *TERT* genetic variants in AA patients and to correlate with prognostic factors such as TL, *TERT* gene expression, and therapeutic response

Our research results demonstrated 26 benign and pathogenic variants in the screened genes that need more functional analysis to confirm their pathogenicity. We also found that most of AA cases had downregulated *TERT* expression (70%) and shortened TL (82.5%); however, they were not significantly related to therapeutic response.

Future studies of larger cohorts of AA cases using more advanced technologies such as whole exome sequencing (WES) are recommended to allow a better understanding of the molecular background of the disease and genotype–phenotype correlation. Additionally, further studies are necessary to confirm the role of telomere length as a prognostic factor in AA.

## Data Availability

All the data generated or analyzed during the current study are included in the article.

## Abbreviations

AA: Aplastic anemia
BM: Bone marrow
CAMT: Congenital amegakaryocytic thrombocytopenia
DC: Dyskeratosis congenita
DEB: Diepoxybutane
FISH: Fluorescent in situ hybridization
HSCT: Hematopoietic stem cell transplantation
IST: Immunosuppressive therapy
MPL: Myeloproliferative leukemia virus oncogene
RI: Reliability index
RQ: Relative quantification
TERC: Telomerase RNA component
TERT: Telomerase reverse transcriptase
TPO: Thrombopoietin
TL: Telomere length
WES: Whole exome sequencing
